# Down-Regulation of GEP100 Causes Increase in E-Cadherin Levels and Inhibits Pancreatic Cancer Cell Invasion

**DOI:** 10.1371/journal.pone.0037854

**Published:** 2012-05-25

**Authors:** Chuan-gao Xie, Shu-mei Wei, Jia-min Chen, Xuan-fu Xu, Jian-ting Cai, Qin-yu Chen, Li-tao Jia

**Affiliations:** 1 Department of Gastroenterology, Second Affiliated Hospital of Zhejiang University College of Medicine, Hangzhou City, Zhejiang Province, China; 2 Department of Pathology, Second Affiliated Hospital of Zhejiang University College of Medicine, Hangzhou City, Zhejiang Province, China; 3 Department of Gastroenterology, Tenth Hospital of Tongji University, Shanghai City, China; Wayne State University School of Medicine, United States of America

## Abstract

**Aims:**

Invasion and metastasis are major reasons for pancreatic cancer death and identifying signaling molecules that are specifically used in tumor invasion is of great significance. The purpose of this study was to elucidate the role of GEP100 in pancreatic cancer cell invasion and metastasis and the corresponding molecular mechanism.

**Methods:**

Stable cell lines with GEP100 knocked-down were established by transfecting GEP100 shRNA vector into PaTu8988 cells and selected by puromycin. qRT-PCR and Western blot were performed to detect gene expression. Matrigel-invasion assay was used to detect cancer cell invasion *in vitro*. Liver metastasis *in vivo* was determined by splenic injection of indicated cell lines followed by spleen resection. Immunofluorescence study was used to detect the intracellular localization of E-cadherin.

**Results:**

We found that the expression level of GEP100 protein was closely related to the invasive ability of a panel of 6 different human pancreatic cancer cell lines. Down-regulation of GEP100 in PaTu8988 cells significantly decreased invasive activity by Matrigel invasion assay, without affecting migration, invasion and viability. The inhibited invasive activity was rescued by over-expression of GEP100 cDNA. *In vivo* study showed that liver metastasis was significantly decreased in the PaTu8988 cells with GEP100 stably knocked-down. In addition, an epithelial-like morphological change, mimicking a mesenchymal to epithelial transition (MET) was induced by GEP100 down-regulation. The expression of E-cadherin protein was increased 2–3 folds accompanied by its redistribution to the cell-cell contacts, while no obvious changes were observed for E-cadherin mRNA. Unexpectedly, the mRNA of Slug was increased by GEP100 knock-down.

**Conclusion:**

These findings provided important evidence that GEP100 plays a significant role in pancreatic cancer invasion through regulating the expression of E-cadherin and the process of MET, indicating the possibility of it becoming a potential therapeutic target against pancreatic cancer.

## Introduction

Pancreatic cancer is the fourth leading cause of cancer death in the United States [1]. Recent data estimated that 43,140 new cases were diagnosed, with approximately 36,800 associated deaths in 2010 [2]. Pancreatic cancer is often diagnosed at the advanced stages with local invasion and remote metastasis, making surgical resection difficult and less effective [3]. Thus, an enormous amount of effort has been made to try to inhibit the invasive activities of carcinoma cells. For the development of therapeutics, it is of great significance to identify the signaling molecules that are specifically used in tumor invasion [4], [5], [6].

Guanine nucleotide-exchange protein 100, GEP100 (also called BRAG2) was identified as one of the guanine nucleotide exchange factors (GEF) that preferentially accelerated guanosine 5-[γ-thio]triphosphate (GTPγS) binding for Arf6 and responsible for its following activation [7]. It has been reported that GEP100 was involved in various biological processes including cell surface receptor expression, cell-cell fusion, adhesion, phagocytosis, apoptosis and angiogenesis [8]–[15]. Recent studies showed that GEP100 played an important role in tumor invasion. GEP100 links epidermal growth factor receptor signaling to Arf6 activation to induce breast cancer and lung cancer invasion [16], [17]. At present, the function of GEP100 in other cancers, including pancreatic cancer, remains unknown.

A process called epithelial to mesenchymal transition (EMT) often occurs during carcinoma progression. During this process, the epithelial cells overcome the physical constraints imposed on them by intracellular junctions and obtain the mesenchymal phenotype and enhanced motility [18]. E-cadherin is a transmembrane protein localized at the adherens junctions of the basolateral surface and plays an important role in epithelial morphology maintenance. Loss of E-cadherin expression and/or function is a well-recognized marker of EMT and promotes pancreatic cancer cell progression and invasion, relating to the poor prognosis of pancreatic cancer [19]–[23]. To date, the effect of GEP100 on EMT is rarely studied.

Therefore, in this study, we investigated the function of GEP100 in the processes of pancreatic cancer cell invasion, metastasis and EMT. We analyzed GEP100 expression in different cell lines and found that the GEP100 expression level was closely related to cell invasive abilities. Matrigel invasion assay *in vitro* and liver metastasis experiment *in vivo* both revealed that down-regulation of GEP100 inhibited invasion and metastasis significantly. The expression of E-cadherin was up-regulated by GEP100 knock-down. Our findings will help further revealing molecular mechanisms used in the pancreatic cancer invasion and metastasis processes.

## Materials and Methods

### Cell Culture and Antibodies

Human pancreatic cancer cell lines BxPC-3, CFPAC-1, SW1990, AsPC-1, Panc-1 and PaTu8988 were all obtained from Chinese Academy of Sciences Cell Bank (Shanghai, China) and cultured in RPMI-1640 (Gibco) containing 10% fetal bovine serum without antibiotics in a 5% CO_2_ incubator at 37°C.

Primary antibodies for Arf6, vimentin, β-actin were purchased from Santa Cruz Biotechnology. Antibodies for GEP100 and E-cadherin C36 were from Sigma and BD Biosciences respectively. All secondary antibodies were from Boster Technology (Wuhan, China).

### Plasmids and Transfection

The pEGFP-GEP100 plasmid encoding the full length of GEP100 cDNA and pSuper-retro-puro-GEP100 plasmid encoding GEP100 shRNA were kind gifts from professor Sabe Hisataka (Hokkaido University, Japan). For shRNA-mediated GEP100 suppression, 8×10^5^ of PaTu8988 cells were transfected with 3 µg of psuper-retro-puro-GEP100 or a plasmid encoding an irrelevant sequence using Lipofectamine 2000 (Invitrogen) for 48 hours. The transfected cells were selected with 1 µg/ml puromycin (Invitrogen) and pools of selected cells were subjected to *in virto* experiments including invasion, wound healing, adhesion and viability assays. For the generation of stable cell lines, the transfected cells were selected with 1 µg/ml puromycin and pools of selected cells were serially diluted in a selection medium. Approximately 20 clones were isolated and tested with Western blot. The clone displaying the highest degree of suppression was used for *in vivo* experiments.

### Viability, Adhesion, Wound Healing and Matrigel Invasion Assays

Cell viabilities were measured with a MTT colorimetric assay kit (Promega) according to the manufacturer’s instructions.

For the adhesion assay, a 35 mm culture dish was coated with 10 µg/ml collagen I (Sigma-aldrich) and then 1×10^5^ cells were seeded. 1 hour later, the dish was washed with PBS gently for 3 times. The number of cells adhered to the dish was counted.

For the wound healing assay, 1×10^6^ cells were seeded into a 35 mm culture dish and allowed to form a monolayer. A wound was made by scratching the monolayer with a 100 µl pipette tip. The cells were cultivated till the wound was covered.

The Matrigel invasion assay was performed using a transwell coated with 25 µg Matrigel (Sigma). 1×10^5^ cells in RPMI-1640 with 1% phosphate buffer saline (PBS) were seeded onto the upper well. As a chemoattractant, RPMI-1640 with 10% FBS was added into the lower compartment. After incubation for 24 hours, cells were fixed in methanol for 15 min and stained with 1% crystal violet (Sangon, China) for 10 min. The cells on the upper surface of the filter were wiped off with a cotton swab and the number of cells that migrated out to the lower surface of the membranes was counted in 10 randomly selected fields.

### RT-PCR Analysis

Total RNA was isolated from cultured cells by Trizol (Invitrogen) and reverse-transcribed by M-MLV Reverse Transcriptase (Promega) using oligo dT primers at 42°C for 60 min. cDNAs were then subjected to 35 cycles of PCR amplification. Primers used were listed below: GEP100, forward primer (5′-GCCTTTAGCAACGATGTCATC-3′) and reverse primer (5′-CACATGGTCCTCATTGGTCTT-3′); Arf6, forward primer (5′-ATGGGGAAGGTGCTATCCAAAATC-3′) and reverse primer (5′-GCAGTCCACTACGAAGATGAGACC-3′); E-cadherin, forward primer (5′-TCCCATCAGCTGCCCAGAAA-3′) and reverse primer (5′-TGACTCCTGTGTTCCTGTTA-3′); Vimentin, forward primer (5′-GACAATGCGTCTCTGGCACGTCTT-3′) and reverse primer (5′-TCTTCTGCCTCCTGCAGGTTCTT-3′); Slug, forward primer (5′-AGATGCATATTCGGACCCAC-3′) and reverse primer (5′-CCTCATGTTTGTGCAGGAGA-3′); Twist, forward primer (5′-CACTGAAAGGAAAGGCATCA-3′) and reverse primer (5′-GGCCAGTTTGATCCCAGTAT-3′); ZEB1, forward primer (5′-AGCAGTGAAAGAGAAGGGAATGC-3′) and reverse primer (5′-GGTCCTCTTCAGGTGCCTCAG-3′); Snail, forward primer (5′-TTCTTCTGCGCTACTGCTGCG-3′) and reverse primer (5′-GGGCAGGTATGGAGAGGAAGA-3′). The primers used for β-actin were purchased from Sangon, China.

### Western Blot Analysis

Cells were lysed in RIPA lysis buffer (150 mM NaCl, 20 mM Tris-HCl pH7.4, 5 mM EDTA, 1% NP-40, 1% Na-deoxycholate, 0.1% SDS, 1 mM PMSF, 20 µg/ml Leupeptin, 20 µg/ml Aprotini, 3 µg/ml Pepstatin A) and the protein concentration was determined using the BCA kit (Keygen, China). Total proteins were fractionated using SDS-PAGE and transferred onto a nitrocellulose membrane. The membranes were blocked in 5% BSA in TBST buffer containing 0.1% Tween 20 and then incubated with indicated primary antibodies overnight at 4°C. HRP-conjugated secondary antibodies were incubated at room temperature (RT) for 1 hour and detected using the enhanced chemiluminesence detection system (Amersham Pharmacia Biotech).

### Immunofluorescence Study

The transfected PaTu8988 cells were washed with warm PBS once and immediately fixed with cold methanol at −20°C for 6 min. The sample was allowed to dry at RT for 30 min and blocked with 2% BSA in PBS for 30 min. The endogenous E-cadherin was recognized with an anti-E-cadherin monoclonal antibody for 1 hour at RT. After 5 washes with 0.5% BSA-PBS, a FITC-labeled anti-mouse secondary antibody (Boster, China) was added and incubated for 30 min at RT. Then the cells were washed with PBS for 5 times again, then mounted with 50% glycerol in PBS and observed under a fluorescence microscope.

### *In vivo* Metastasis Assay

A liver metastasis assay was performed as described previously [24], [25] with modifications. Briefly, Balb/c nude mice (5–6 weeks) were divided into 3 groups with 12 for each: a control group receiving the PaTu8988 cells, a scramble group receiving the cells knocked-down with sramble shRNA and an experimental group receiving the cells with GEP100 stably knocked-down. Mice were anaesthetized with chloral hydrate. The abdominal cavity was exposed and 5×10^6^ indicated cells in 0.1 ml PBS were injected into the spleen tissue through a 27-gauge needle. The injection site was pressed slightly with saline cotton for 5 min followed by the ligation of the left gastric artery and splenic artery. Then the spleen was resected. 4 weeks later, the liver was surgically removed and fixed with 10% formalin overnight. The liver was cut into 2 mm slices and 5 sections from approximately the same position for each liver were estimated under a microscope. The protocols used for all animal experiments in this study were approved by the Animal Research Committee of Zhejiang University.

### Statistical Analysis

The experiments and assays were performed at least three times. Statistical significance was assumed if *P*≤0.05 by T-test.

## Results

### Correlation between GEP100 Expression and Pancreatic Cancer Cell Invasive Ability

**Figure 1:**
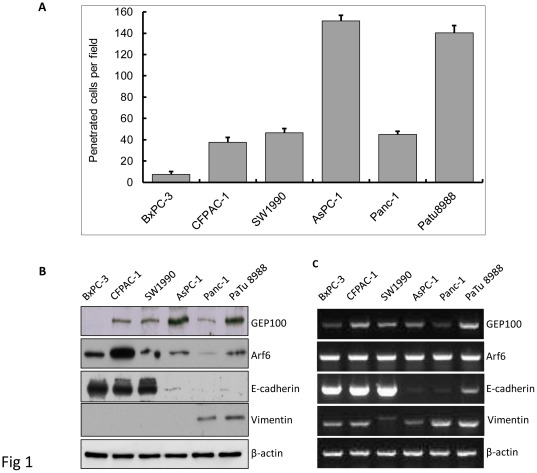
GEP100 expression correlates closely with pancreatic cancer cell invasive ability. (A) Invasive abilities of a panel of 6 pancreatic cancer cell lines were analyzed by Matrigel invasion assay for 24 hours. AsPc-1 and PaTu8988 showed high invasive abilities. (B) Western blot analysis of the expression of GEP100, Arf6, E-cadherin and vimentin protein in different cell lines. AsPc-1 and PaTu8988 showed the strongest expression of GEP100 protein, followed by SW1990, CFPAC-1, Panc-1 and BxPC-3, demonstrating a close relationship between GEP100 expression level and cell invasive ability. No obvious relationship between the expression of Arf6 protein and invasive ability was detected. E-cadherin expression could easily be detected in the low-invasive cell lines, but only a very weak expression was detected in the highly-invasive cell lines. Vimentin was expressed in the highly-invasive cell lines. (C) RT-PCR analysis of the expression of GEP100, Arf6, E-cadherin and vimentin mRNA. The expression of GEP100 mRNA could be detected in all the six cell lines and no obvious correlation with the invasive ability was found. This was the same case for Arf6 and vimentin mRNA. E-cadherin mRNA showed a close association with the invasive ability of different cell lines. β-actin mRNA was used as a control.

The invasive ability of a panel of 6 different human pancreatic cancer cell lines was examined by the Matrigel invasion assay as shown in Fig. 1A. The cell lines in this group were derived from both the primary (BxPC3, Panc-1, and SW1990) and metastatic (AsPC-1, CFPAC-1, and Patu8988) sites [26], [27]. These cell lines showed a continuum of different invasive abilities. The AsPc-1 and PaTu8988 cells showed the highest invasive abilities, followed by the SW1990, CFPAC-1 and Panc-1 cells. The BxPC-3 cell was the weakest one. The expression level of GEP100 protein was closely correlated with the invasive ability of this panel of cell lines, with the AsPc-1 and PaTu8988 cells showing the strongest expression, followed by the SW1990, CFPAC-1 and Panc-1 cells, while hardly detected in the BxPC-3 cell line. No obvious relationship between the expression of the Arf6 protein and invasive ability was detected. We also determined the expression levels for the proteins associated with epithelial (E-cadherin) or mesenchymal (Vimentin) phenotype. E-cadherin expression could easily be detected in the low-invasive cell lines, but only a very weak expression was detected in the highly-invasive cell lines. Vimentin was expressed in the highly-invasive cell line (Fig. 1B).

The expression of GEP100 mRNA could be detected in all the 6 cell lines and no obvious correlation with the invasive ability was found. This was the same for Arf6 and vimentin mRNA. E-cadherin mRNA level showed a close association with invasive ability of different cell line (Fig. 1C).

### Down-regulation of GEP100 Decreased the Invasive Ability of Pancreatic Cancer Cells

**Figure 2:**
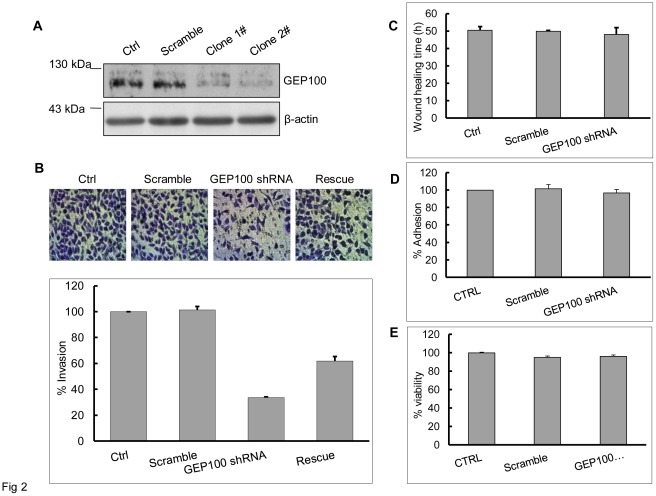
Down-regulation of GEP100 inhibits pancreatic cancer cell invasion. (A) A stable cell line with GEP100 knocked-down was obtained by transfecting psuper-retro-puro-GEP100 into PaTu8988 cells and selected by 1.5 µg/ml of puromycin. Down-regulation of GEP100 was confirmed by Western blot. β-actin was used as a control. (B) Invasion assay showed that GEP100 down-regulation decreased the number of cells penetrated through Matrigel-coated membrane. The data were presented and graphed as percentages calculated by normalizing values obtained for the untreated cells as 100%. GEP100 knock-down decreased the number of cells penetrated through Matrigel to about 35% compared with the control group. Re-expression of GEP100 in shRNA treated cells restored the ability of invasion to about 60%. (C, D, E) GEP100 down-regulation showed no significant effects on cell migration, invasion and viability. Ctrl, control.

To evaluate whether GEP100 contributes to cell invasion, we chose the highly invasive PaTu8988 cells and down-regulated the GEP100 expression with shRNA. More than 80% inhibition of GEP100 protein synthesis was confirmed by Western blot in two clones (Fig. 2A). Clone 1# was chosen for the invasion assay. The Matrigel invasion assay showed that GEP100 knock-down decreased the number of cells penetrating through Matrigel to about 35% compared with the control group (Fig. 2B). Re-expression of GEP100 restored the ability of invasion to about 60%. Migratory activity was evaluated by the wound healing assay and wound healing time periods of three groups were shown. GEP100 knock-down only slightly inhibited the migration (Fig. 2C) with no statistical significance. On the other hand, GEP100 knock-down did not inhibit cell adhesion onto collagen (Fig. 2D). Under the above conditions, cell viability was not affected (Fig. 2E).

### Down-regulation of GEP100 Decreased Liver Metastasis of Pancreatic Cancer Cells in Balb/c Nude Mice

**Figure 3:**
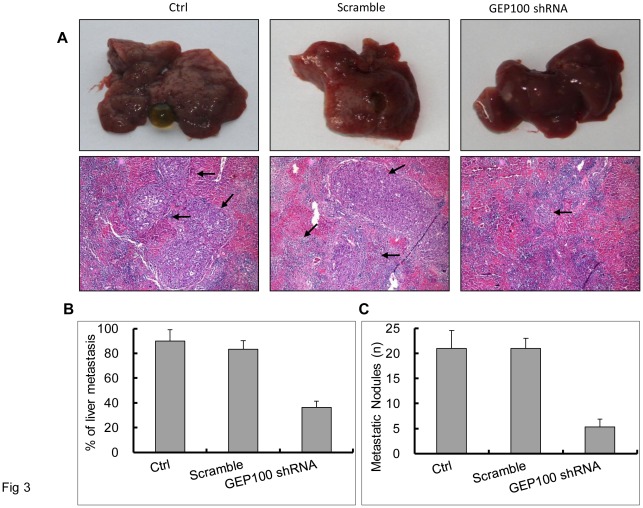
GEP100 down-regulation inhibits liver metastasis of pancreatic cancer cells in the Balb/c nude mice with splenic injection. (A) Macroscopic findings of the resected liver. Stable PaTu8988 cells as indicated were splenicly injected followed by spleen resection. 4 weeks later, livers were removed for observation. Most of the livers from the control and scramble shRNA groups became small and rough, with large whitened areas seen on the surface. For the GEP100 shRNA-injected group, only small spots were found at some of the liver surface. Metastasis was confirmed under a microscope and the sites of metastasis were indicated by arrows (hematoxylin and eosin, x100). (B) Percentages of liver metastasis in the Balb/c nude mice were listed. A statistically significant difference was obtained between control and experimental group (*P*≤0.05). (C) Average numbers of metastatic nodules in livers were calculated and graphed. Briefly, after a fixation with 10% formalin overnight, the liver was cut into 2 mm slices and 5 sections from approximately the same positions for each liver were estimated under microscope. A statistically significant difference was obtained between control and experimental group (*P*≤0.05). Ctrl, control.

Next, we examined whether knock-down of GEP100 blocks liver metastasis of tumor cells in mice. We injected PaTu8988 or PaTu8988/scramble shRNA or PaTu8988/GEP100 shRNA cells into the spleens of Balb/c nude mice. Twelve mice from each group were sacrificed after 4 weeks post injection. For the control and scramble shRNA-injected groups, the liver became small and rough, with whitened areas seen on the surface. For the GEP100 shRNA-injected group, small spots were also found at the liver surface. Hepatic metastasis showing poorly differentiated nests of cells was confirmed microscopically in 9 of 10 mice (90%) in the control group and 10 of 12 mice (83%) in the sramble group, while only 4 of 11 mice (36%) was found in the experimental group (Fig. 3). A significant inhibitory effect on liver metastasis was observed (*P*≤0.05). The numbers of metastatic nodules were calculated and averaged. Comparing with the control and the scramble groups, GEP100 down-regulation decreased the metastatic nodules significantly (*P*≤0.05).

### E-cadherin Expression was Up-regulated and Redistributed to the Cell-cell Contact by GEP100 Down-regulation

**Figure 4:**
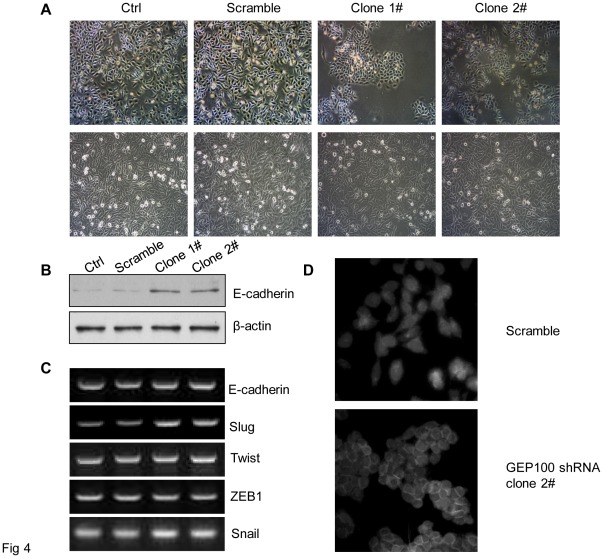
GEP100 down-regulation induces epithelial change of cancer cells and up-regulation of E-cadherin protein. (A) A mesenchymal to epithelial change was observed in the cells stably knocked-down for GEP100. The upper and lower panels showed the morphological appearances at a low cell density and when the cells reached confluence, respectively. Comparing with the control and scramble groups, the cell in the experimental group became epithelial-like and adhesive. (B) Western blot showed that the expression level of E-cadherin protein was increased about 3-fold in the experimental group compared with the control group. (C) RT-PCR analysis of E-cadherin mRNA and its transcription regulators. The expression of E-cadherin mRNA was not affected by GEP100 down-regulation. An increase of Slug mRNA was found in the GEP100 knocked-down cells, while there was no change for Twist, ZEB1 and Snail. (D) Immunofluorescence staining of E-cadherin. Comparing with the scramble group, GEP100 down-regulation redistributed the E-cadherin into the cell-cell contacts.

Finally, we investigated the possible mechanisms involved in the invasion inhibition of pancreatic cancer cells by GEP100 down-regulation. We found that down-regulation of GEP100 caused a morphological change of PaTu8988 cells from mesenchymal type to epithelial-like (Fig. 4A). And correspondingly, the expression of E-cadherin protein, which is an epithelial marker, was increased about 3-fold compared with the control group (Fig. 4B). The expression of E-cadherin mRNA was also examined, but no significant changes could be detected (Fig. 4C). Next, we further determined the intracellular localization of E-cadherin protein. Immunofluorescence study showed that in GEP100 knocked-down cells, E-cadherin was concentrated at the cell-cell contact (Fig. 4D). Although no significant change was found at the mRNA level of E-cadherin, we still examined the expression of several main transcription factors for E-cadherin. Interestingly, an increase of Slug was found in the GEP100 knocked-down cells, while there was no change for Twist, ZEB1, Snail (Fig. 4C).

## Discussion

The current results demonstrated for the first time that GEP100 plays a pivotal role in pancreatic cancer cell invasion. Down-regulation of GEP100 significantly inhibited the invasive abilities of pancreatic cancer cells possibly through the up-regulation of the expression of E-cadherin and restoration of the epithelial phenotype. GEP100 might be a potential molecular target in pancreatic cancer gene therapy.

In this study, first, we demonstrated that GEP100 was expressed in the pancreatic cancer cells. GEP100 belongs to the BRAG family of Arf GEF, consisting of three isoforms, BRAG1/IQsec2, BRAG2/IQsec1/GEP100, and BRAG2/IQsec3/synArfGEF [28]. The expression of BARG2/GEP100 is ubiquitous [7]. Within the panel of 6 pancreatic cancer cell lines we used, BxPC-3, SW1990 were derived from primary tumors and Panc-1 was derived from invasive intraductal extension of primary tumor, while the other 3 were all derived from metastatic sites [26], [27]. AsPc-1 and PaTu8988 showed the strongest expression of GEP100, followed by SW1990, CFPAC-1 and Panc-1, while the primary tumor of BxPC-3 cells showed the weakest. The expression of GEP100 protein is closely related to the invasive ability of each cell line, suggesting that GEP100 might be involved in pancreatic cancer cell invasion.

We further found that GEP100 down-regulation with shRNA significantly inhibited the Matrigel invasion ability of PaTu8988 cells, but had no effect on the cell viability, migration and adhesion, indicating that GEP100 is particularly responsible for the invasive ability of cells. *In vivo* experiment also showed that GEP100 knock-down significantly inhibited the liver metastasis of pancreatic cancer cells in Balb/c nude mice. This result was in line with previous data demonstrating that in breast cancer cells GEP100 was highly expressed in the invasive ones and that GEP100 down-regulation inhibited cell invasion and lung metastasis [16], [17]. These results indicated that GEP100 is not only a target for breast cancer, it might be a general mechanism used by other cancer types.

Several other biological functions for GEP100 have been reported. In HeLa cells, GEP100 regulated cell adhesion through controlling endocytosis and recycling of integrin β [9]. In a liver carcinoma cell line HepG2, GEP100 directly interacted with α-catenin and played a role in actin cytoskeleton remodeling [10]. In myoblasts and macrophages, GEP100 was involved in cell-cell fusion [11]. It has also been reported to take part in the regulation of nucleolar architecture and phagocytosis [12], [13]. In this study, we did not observe any significant effect of GEP100 on cell adhesion to collagen matrix or migration activity. Since there is not any report on the role of GEP100 in pancreatic cancer cells, this may be due to the difference in cell type.

Unlike the previous report on a close relation between Arf6 expression and breast cancer cell invasive ability [29], we failed to detect that in the panel of pancreatic cancer cell lines we used. The above mentioned biological functions of GEP100 have been found to be dependent on the activation of Arf6, but it is believed that GEP100 is a multifunctional protein that regulates cellular functions in both an Arf-dependent and -independent manner. For example, GEP100 was involved in apoptotic cell death independent of Arf6 activity [12]. Further studies including determination of the activation status of Arf6 will be necessary to reveal the role of Arf6 in the process of pancreatic cancer cell invasion.

We found GEP100 knock-down caused a morphological change of cells from mesenchymal to epithelial phenotype. In pancreatic cancer, shifting of their epithelial features toward a mesenchymal phenotype enhances cell motility and is considered to be a prerequisite for tumor invasion. E-cadherin is the best characterized molecular marker in epithelial cells and localized at the adherens junctions. Loss of E-cadherin expression and/or function is a well-recognized marker of EMT and promotes invasion [18]. Therefore we examined the expression of E-cadherin protein. GEP100 shRNAs treatment increased E-cadherin expression level 2 to 3 -fold. This is consistent with Hiroi’s report showing that in HepG2 cell, down-regulation of GEP100 increased the expression of E-cadherin [10]. In addition, they also demonstrated that GEP100 interacted with α-catenin.

E-cadherin expression could be regulated by multiple mechanisms including promoter methylation, transcriptional regulation by the transcription factors including Snail, Slug, Twist, Zeb-1, Sip1, and proper intracellular localization [30], [31]. Among the transcription factors we examined, the expression of slug was increased by the down-regulation of GEP100, which was unexpected. Slug is a member of the Snail superfamily and first identified as a development protein critical for neural crest formation in chick embryos [32]. Slug is inversely correlated with E-cadherin expression and is a critical EMT-promoting factor in many tumor types [33], [34]. Recent study showed that Slug expression was not always associated with E-cadherin down-regulation [35]. It was clearly shown that Slug expression was increased in pancreatic cancer compared with the surrounding parenchyma. However, in the 36 cases of ductal adenocarcinoma analyzed, no obvious relation was found between E-cadherin and Slug expression [36]. In this study we found that GEP100 down-regulation induced an increased E-cadherin expression as well as increased Slug expression. One interpretation is that the E-cadherin protein expression is not dependent on Slug in this cell type and indeed the mRNA of E-cadherin was not changed. Although the expression of Slug is associated with many tumor prognosis, It is not clear yet how Slug itself is regulated. Our data indicated that GEP100 might be involved in the regulation of Slug expression.

A proper localization of E-cadherin is also critical for its proper function. Cell-surface E-cadherin in epithelial cells is partially internalized and recycled back to the plasma membrane by multiple mechanisms including clathrin-dependent, caveolae-dependent, lipid-raft mediated pathways or macropinocytosis [31]. In epithelial junctions, the dynamics of E-cadherin was also intimately regulated by the ARF proteins [37], [38]. Arf6 GTPase is crucial for E-cadherin endocytosis and recycling. It has been shown that expression of an inactive Arf6T27N protein blocks HGF-induced internalization of E-cadherin, whereas expression of a constitutive active form of Arf6Q67L causes disassembly of adherens junctions [39]. In HepG2 cells, GEP100 depletion caused inactivation of Arf6 followed by impaired internalization of E-cadherin and its accumulation at the plasmam membrane [10]. With immunofluorescence study, we found that GEP100 knock-down increased the accumulation of E-cadherin to adherens junctions. We speculate that GEP100 up-regulated the expression of E-cadherin through inhibiting its endocytosis and the following degradation. Further investigation will be necessary to clarify this issue.

In summary, our results presented experimental evidence that GEP100 expression correlated with the invasive ability of pancreatic cancer cells and could be considered as a new target for developing therapeutics to prevent pancreatic cancer cell invasion.
